# Changes in Socioeconomic Inequality in Indonesian Children’s Cognitive Function from 2000 to 2007: A Decomposition Analysis

**DOI:** 10.1371/journal.pone.0078809

**Published:** 2013-10-30

**Authors:** Amelia Maika, Murthy N. Mittinty, Sally Brinkman, Sam Harper, Elan Satriawan, John W. Lynch

**Affiliations:** 1 School of Population Health, University of Adelaide, Adelaide, South Australia, Australia; 2 Adelaide Branch of the Telethon Institute for Child Health Research, Adelaide, South Australia, Australia; 3 Department of Epidemiology, Biostatistics & Occupational Health, McGill University, Montreal, Quebec, Canada; 4 Faculty of Economics and Business, Gadjah Mada University, Yogyakarta, Indonesia; 5 The National Team for Accelerating Poverty Reduction (TNP2K), Jakarta Pusat, Indonesia; University Children's Hospital Tuebingen, Germany

## Abstract

**Background:**

Measuring social inequalities in health is common; however, research examining inequalities in child cognitive function is more limited. We investigated household expenditure-related inequality in children’s cognitive function in Indonesia in 2000 and 2007, the contributors to inequality in both time periods, and changes in the contributors to cognitive function inequalities between the periods.

**Methods:**

Data from the 2000 and 2007 round of the Indonesian Family Life Survey (IFLS) were used. Study participants were children aged 7–14 years (n = 6179 and n = 6680 in 2000 and 2007, respectively). The relative concentration index (RCI) was used to measure the magnitude of inequality. Contribution of various contributors to inequality was estimated by decomposing the concentration index in 2000 and 2007. Oaxaca-type decomposition was used to estimate changes in contributors to inequality between 2000 and 2007.

**Results:**

Expenditure inequality decreased by 45% from an RCI = 0.29 (95% CI 0.22 to 0.36) in 2000 to 0.16 (95% CI 0.13 to 0.20) in 2007 but the burden of poorer cognitive function was higher among the disadvantaged in both years. The largest contributors to inequality in child cognitive function were inequalities in per capita expenditure, use of improved sanitation and maternal high school attendance. Changes in maternal high school participation (27%), use of improved sanitation (25%) and per capita expenditures (18%) were largely responsible for the decreasing inequality in children’s cognitive function between 2000 and 2007.

**Conclusions:**

Government policy to increase basic education coverage for women along with economic growth may have influenced gains in children’s cognitive function and reductions in inequalities in Indonesia.

## Introduction

In 1970 Indonesia was among the poorest countries in the world with 60% of the population living in absolute poverty [1]. In the decade from 2003, the poverty rate in Indonesia decreased from 17% to 12% and economic growth in the past decade has moved Indonesia from a low to a middle-income country [2]. Despite this overall progress, regional and socioeconomic disparities within the country are still evident, driven by inequalities in economic, infrastructure and human resources [3], [4]. For example, the mean years of schooling for the household head in poor families was 5 compared to 8 years for non-poor families [4]. Fewer than half of the households in 2011 had access to safe drinking water and only about 56% had access to a latrine connected to septic tank or a composting toilet [5].

Measuring inequalities in health related outcomes is relatively common [6]–[8], but research examining inequalities in children’s development is more limited. Children under five living in poorer socioeconomic circumstances in low and middle income countries are often exposed to a multitude of risk factors such as poverty, malnutrition, poor housing conditions and sanitation that influence their opportunities for healthy child development [9], [10]. There is growing interest in the influences of children’s health, learning and well-being, on their later school readiness, academic achievement and labor force participation [11]. Cognitive function is an important aspect of healthy child development as it has both short and longer terms effects. Higher cognitive function is associated with better academic achievement [12], [13] physical and mental health [14]–[16] and in the long-term economic outcomes such as higher occupational status, earnings and may influence national economic performance [17], [18]. Early life social disadvantage has been associated with poorer cognitive outcomes and neurodevelopment in richer and poorer countries [19]–[24]. Among school aged children, inequality in early life socioeconomic circumstances also contributes to inequality in children’s cognitive outcomes as measured through literacy [25], [26] and math scores [26], [27].

The aim of the current study was to quantify household expenditure-related inequality in Indonesian children’s cognitive function in 2000 and 2007. We also investigated the contributions of child, parental and household characteristics to inequality in both periods and changes in contributions to children’s cognitive function inequalities between 2000 and 2007.

## Methods

### Ethics statement

This research has been approved by Human Research Ethics Committee the University of Adelaide.

### Data

We used data from the 2000 and 2007 round of the Indonesia Family Life Survey (IFLS), which is an ongoing longitudinal survey in Indonesia. IFLS was conducted in 1993, 1997, 2000 and 2007. IFLS provided extensive information about socioeconomic, behavior and health related outcomes at household and individual levels, as well as information about public facilities at the community level. IFLS used multi-stage sampling. Stratified sampling was used to select province, which covers 13 out of 27 provinces in 1993. Random sampling was used to select households within these provinces. The sample of households represented 83% of the Indonesian population living in the 13 provinces in 1993 [27]. In this study, we used data from the third (2000) and fourth (2007) round of the IFLS [28], [29]. We selected participants aged 7 to 14 years who were interviewed for cognitive assessment in 2000 (n = 6179) and aged 7 to 14 in 2007 (n = 6680). The response rate for this cognitive test was 96% for each year. The data was analyzed as a repeated cross sectional study and was weighted using cross-sectional person sampling weights provided in the IFLS datasets for 2000 and 2007.

**Cognitive function.** Cognitive function was measured using a subset from the Raven’s Progressive Matrices, comprising 12 shapes with a missing part where children selected the correct part to complete the shape [30].Each correct answer was coded 1 or 0 otherwise and scores combined as the total raw score. The distributions of the total raw scores were skewed towards the left tail at all ages, and as expected also increased with age. Because scores were highly skewed, we calculated the mean and the variance by taking into account the range, median and the sample size using the formula from Hozo, et al [31] and used the estimated mean and standard deviation to create an age specific z-score.

**Per capita expenditure.** We used the log of per capita expenditure which was constructed from the monthly total household expenditures divided by the number of household members [32].

**Covariates.** A range of child, parental and household characteristics was selected *a priori* as contributors to inequality in children’s cognitive function. Children’s characteristics included gender and whether the child was currently attending school [30], [33], [34]. Parental characteristics were measured separately for father and mother including education, employment and mental health [20]–[22], [35], [36]. Parental education was measured as the highest level of education attended and was recoded in three categories, none or primary, high school, or university. Parental primary employment was defined whether parents were working in the past week, categorized as “yes” and “no”. Parental mental health was measured using the short version of the Centre for Epidemiology Studies Depression Scale (CES-D) [37]. For this study we used mental health as a continuous variable from total scores of the CES-D measure, where higher score is associated with poorer mental health symptoms. Household characteristics included whether the household had electricity, used an improved drinking water source (defined as piped water, electric or hand pumps boreholes and bottled water) and improved sanitation (defined as toilet with septic tank) [35], [38], [39].Residential area included whether the household was in a rural area and province of residence, categorized as Java Bali or otherwise.

### Missing data

In 2000, of the 6179 children, 2023 children had missing data in one or more variables of interest (exposure, outcome or covariate) leaving 4156 children with complete information. In 2007, of the 6680 children, 2389 children had missing data in one or more variable of interest and 4291 children had complete information. We performed Multiple Imputation by Chained Equation (MICE) procedure in STATA to impute all the missing variables under the assumption that the data are missing at random [33]. We generated a total of twenty imputed datasets using fifty cycles of regression switching. Children who do not have either one or both parents, due to death or were not residing in the same household were excluded from the imputation analysis. Imputations were conducted for each study year separately. Multiple imputation was only used for the estimation of the RCI not in the decomposition. That is because there are currently no methods for combining the estimates in the decomposition part of the analysis.

## Analysis

### The magnitude of the inequality in children’s cognitive function

The concentration curve is a graphical illustration of the magnitude of the inequality in children’s cognitive function. The relative concentration curve can be drawn by plotting the cumulative share of the population ranked by the log of per capita expenditures (starting from the lowest to the highest) on the x-axis, against the cumulative share of cognitive function z-score on the*y-*axis. We used the relative concentration index (RCI) to calculate the magnitude of the inequality, defined as twice the area between the curve and the line of equality [6], [40]. The RCI can be written as



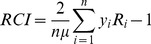
(1)Where *μ* is the mean of cognitive function z-score (*y*), *R_i_* is the relative rank of the *i*th individual in the per capita expenditures distribution. The RCI normally takes value -1 to 1 with a value of zero indicating no inequality. However, the value is not bounded when the *y* takes negative and positive values [41]. In our study, *y* is a positive outcome (higher score is associated with a better cognitive function), so positive values of concentration index indicate children with higher cognitive function are concentrated among the non-poor and vice versa. We applied the Delta method to estimate the standard errors (SE) of the RCI [41] and then used the estimated SE to calculate confidence interval. For imputed data we estimate RCI and SE in each of the twenty datasets and then use Rubin’s rules for combining these results [42].

### Decomposition of contributors to inequality in children’s cognitive function

We used decomposition analysis to estimate contributions to inequality in children’s cognitive function for each year [6], [41]. In decomposition analysis a set of *k* contributors (*x_k_*) is regressed on continuous *y* in a linear regression model (Equation 2),where 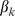 are the coefficients and *ε_i_* is an error term.



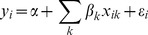
(2)Given the relationship between *y_i_ and x_ik_* in Equation 2, the RCI is estimated as the sum of the relative concentration index of the determinants weighted by the elasticity (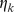) of *y* with respect to each determinant. The formula can be written as



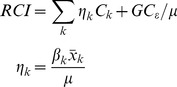
(3)where 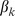 is the estimated coefficient of *k* contributor, 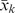 is the mean of *k, μ* is the mean of *y, C_k_* is the relative concentration index for *k* contributor, and *GC_ε_* is the generalized concentration index for the error term. The contribution of each factor is a function of the elasticity of cognitive function with respect to the particular contributor and the degree of per capita expenditures-related inequality. Therefore, in order to have a large contribution to the total inequality, a factor should have either large elasticity or large relative concentration index (*C_k_*) or both.

For estimating the uncertainty in the decomposition we used Markov chain Monte Carlo (MCMC) simulation method [43].This was chosen over bootstrap methods because it allows use of the survey weights without requiring any additional computational complexity. In MCMC we used the Gibbs re-sampling method. The 95% confidence interval was calculated using the equal tail method. The equal tail interval runs from 2.5^th^ percentile and 97.5^th^ percentile of the posterior distributions [44].The decomposition analysis is limited to complete case because the methodology for estimating the SE of percent contribution of each determinant is not yet available, which in-turn limits the use of Rubin’s rule for combining the imputed data.

### Decomposition of changes in the inequality in children’s cognitive function

We also examined changes in per capita expenditure inequality in cognitive function between 2000 and 2007. We used the Oaxaca-type decomposition to measure changes in inequality in the contributors to cognitive function inequality and changes in the elasticity of cognitive function with respect to these contributors [6], [41].The formula can be written as



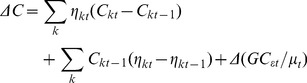
(4)Where 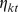 is the elasticity of *k* contributor at time *t* and *C_kt_* is the relative concentration index of *k* at time *t.* Similar to decomposition analysis, the decomposition of change is also restricted to complete case.

## Results

Table 1 shows the summary statistics of cognitive function and its contributors. The average child cognitive function z-score increased from 0.24 (SD 0.79) in 2000 to 0.34 (SD 0.66) in 2007. Of the 4156 children in 2000, 51% were males and 91% of them were still attending school. In 2007, of the 4251 children 95% were still attending school. In terms of parental education, the mothers had lower education than the fathers in both years. In 2000, 69% of mothers had no or primary education, whereas in 2007 this had dropped to 50%. There was also improvement in fathers ‘education, 60% of fathers had no or primary education in 2000 down to 45% in 2007. The average log per capita expenditures was 11.93 *rupiah* (SD 0.70) in 2000 and 12.82 *rupiah* (SD 0.65) in 2007, which is equivalent to an increase from approximately 16 to 37 USD per month. In 2000, the proportion of households that had electricity was 89%, used an improved drinking water source was 51% and used improved sanitation was 44%, whereas in 2007, the proportion was 96%, 55% and 64%, respectively.

**Table 1:** Summary statistics for cognitive function and its contributors using complete case analysis, 2000 and 2007.

	2000	2007
	n = 4156	n = 4291
	% or Mean (SD)	% or Mean (SD)
**Outcome**		
cognitive function (z-score)	0.24 (0.79)	0.34 (0.66)
**Children characteristics**		
Age	10.5 (2.28)	10.2 (2.31)
Gender		
Male	51	52
Female	49	48
Primary activity		
-attending school	91	95
**Parental characteristics**		
Father education		
- none or primary	60	45
- high school	32	44
-university	8	11
Mother education		
-none or primary	69	50
-high school	26	42
-university	5	7
Father primary activity		
-working	93	95
-others	7	5
Mother primary activity		
-working	53	49
-others	47	51
Mental health		
-Father	2.03 (2.98)	3.29 (3.18)
-Mother	2.56 (3.50)	3.53 (3.48)
**Household characteristics**		
Residential area		
-Living in rural	57	49
-Living in urban	43	51
-Living in Java or Bali	58	59
-Living in outside Java or Bali	42	41
Log per capita expenditures	11.93 (0.70)	12.82 (0.65)
Has electricity	89	96
Improved drinking water	51	55
Improved sanitation	44	64

**Figure 1:**
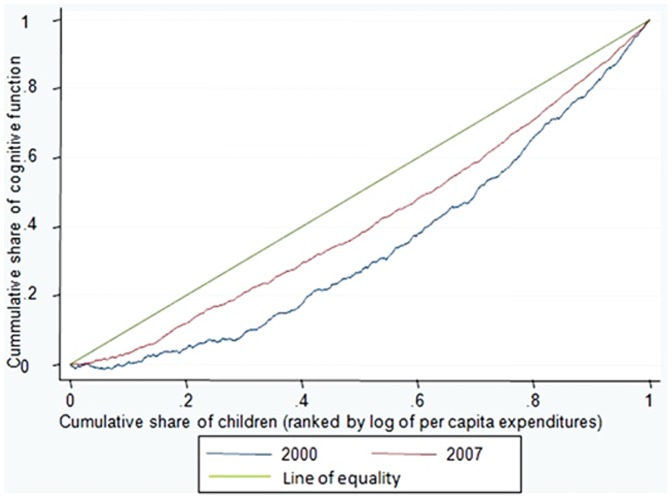
Relative concentration curves for inequality in children’s cognitive function using complete cases analysis, Indonesia 2000 and 2007.

The relative concentration curves for inequality in children’s cognitive function in 2000 and 2007 based on complete case analysis are shown in Figure 1. Using complete case analysis, the RCI in 2000 was 0.29 (95% CI 0.22–0.36) and was 0.16 (95% CI 0.13–0.20) in 2007, showing the burden of poorer cognitive function was higher among the disadvantaged in both years. Table 2 shows the comparison of RCI and their confidence interval for 2000 and 2007 between complete case analysis and multiple imputation analysis. The magnitude of the RCI from multiple imputation analysis were 0.3and 0.4 higher than using complete case analysis, for 2000 and 2007 respectively,

**Table 2:** Comparison between complete case analysis and multiple imputation analysis.

	2000		2007	
	Sample N = 6179	RCI (95% CI)	Sample N = 6680	RCI (95% CI)
Complete case	4156	0.29 (0.22–0.36)	4291	0.16 (0.13–0.20)
Multiple imputation	5079	0.32 (0.24–0.41)	5560	0.20 (0.15–0.25)

Table 3 shows the decomposition of the inequality in cognitive function for 2000 and 2007 ranked by the contribution in 2000. The *β* coefficient shows the association between cognitive function and each contributor. Being male, still attending school, living with a parent who had higher education, and living in Java-Bali were all associated with higher cognitive function scores. Whereas higher parental mental health score and living in a rural area are associated with a lower cognitive function score. The elasticity shows how sensitive cognitive function is to each contributor. The largest elasticity was the log of per capita expenditure (3.38 in 2000 and 3.09 in 2007). The concentration index shows the magnitude of inequality in cognitive function with respect to each contributor. The concentration indices for parental education were all positive, indicating parents with higher education were more concentrated among the higher economic groups. For example, in 2000 the concentration index for the father’s university attendance was 0.67 and was 0.69 for mothers. The concentration indices for parental mental health were negative, indicating that parents with higher mental health scores were more concentrated among the higher economic groups.

**Table 3:** Decomposition of inequality in children's cognitive function ranked by contribution in 2000.

	2000					
Contributors	β	elasticity	concentration	contribution	95% CI	
			index		lower	upper
Log per capita expenditures	0.08	3.38	0.03	37%	19%	55%
Use improved sanitation	0.14	0.21	0.26	18%	11%	26%
Mother attended high school	0.18	0.16	0.33	18%	11%	24%
Father attended university	0.12	0.03	0.67	7%	0%	14%
Mother attended university	0.17	0.03	0.69	7%	1%	12%
Has electricity	0.2	0.65	0.03	6%	4%	9%
Living in rural	–0.07	–0.16	–0.1	6%	2%	9%
Child is attending school	0.17	0.56	0.02	3%	2%	5%
Living in Java/Bali	0.13	0.37	0.02	3%	1%	4%
Father's mental health scores	–0.01	–0.04	–0.06	1%	0%	2%
Mother's mental health scores	–0.01	–0.06	–0.03	1%	2%	9%
Father attended high school	0	0	0.2	0%	–4%	5%
Father is working	0.04	0.13	0.01	0%	0%	1%
Mother is working	0.01	0.02	0.01	0%	0%	0%
Use improved drinking water	0	0	0.16	0%	–5%	5%
Child is male	0.1	0.19	–0.02	–1%	–2%	–1%
Residual		0	0	–5%		
	2007					
Contributors	β	elasticity	concentration	contribution	95% CI	
			index		lower	upper
Log per capita expenditures	0.1	3.09	0.03	52%	33%	70%
Use improved sanitation	0.09	0.14	0.13	12%	6%	18%
Mother attended high school	0.1	0.09	0.16	9%	4%	13%
Father attended university	0.1	0.02	0.57	9%	2%	15%
Mother attended university	0.07	0.01	0.64	5%	–2%	11%
Has electricity	0.25	0.6	0.01	5%	3%	70%
Living in rural	–0.03	–0.05	–0.09	3%	–1%	7%
Child is attending school	0.09	0.22	0.02	2%	0%	4%
Living in Java/Bali	0.14	0.29	0	0%	0%	0%
Father's mental health scores	–0.01	–0.05	–0.03	1%	0%	2%
Mother's mental health scores	0	–0.03	–0.03	1%	0%	2%
Father attended high school	0.04	0.04	0.11	3%	0%	6%
Father is working	–0.13	–0.31	0.01	–2%	–3%	–1%
Mother is working	0.05	0.07	0.07	3%	1%	5%
Use improved drinking water	0.04	0.05	0.09	3%	0%	6%
Child is male	0.05	0.06	–0.01	0%	–1%	0%
Residual		0	0	–3%		

The largest contribution to inequality in cognitive function was inequality in per capita expenditures, accounting for 37% and 52% of the total inequality in 2000 and 2007, respectively. Inequality in using improved sanitation accounted for 18% (in 2000) and 12% (in 2007) of the total inequality in children’s cognitive function. As an important determinant of child cognitive function, parental education disproportionately contributed to the total inequality with maternal high school attendance having the largest contribution in 2000 (18%) and having equal contribution with fathers’ university attendance in 2007 (9%). Although residential location made a very small contribution to the total inequality, children residing in rural areas or outside Java-Bali had poorer cognitive function compared to their urban peers or those children residing in Java-Bali.

There was 0.13 (45%) decrease in inequality in children’s cognitive function between 2000 and 2007. Table 4 shows the decomposition for change in inequality in children’s cognitive function. The first column shows changes in the magnitude of inequality in the contributors and the second column shows changes in the elasticity of the cognitive function with respect to these contributors. The total change for each determinant and the percent change are presented in the last two columns. Overall, changing inequalities and changing elasticities contributed equally to the reduction in inequality in cognitive function. Although inequality in per capita expenditures accounted for the largest contribution to the total inequality for each year, it only contributed 18% to decreasing inequality. Changes in maternal participation in high school (27%), use of improved sanitation (25%) and increases in per capita expenditures were largely responsible for changes in inequality in children’s cognitive function.

**Table 4:** Oaxaca-type decomposition for change in children's cognitive function inequality, 2000–2007.

Contributors	change in inequality	change in elasticity	total	%
Mother attended high school	–0.02	–0.02	–0.04	27%
Use improved sanitation	–0.02	–0.02	–0.03	25%
Log per capita expenditures	–0.02	–0.01	–0.02	18%
Living in rural	0.00	–0.01	–0.01	8%
Mother attended university	0.00	–0.01	–0.01	8%
Has electricity	–0.01	0.00	–0.01	7%
Father attended university	0.00	–0.01	–0.01	5%
Living in Java/Bali	–0.01	0.00	–0.01	5%
Child is attending school	0.00	–0.01	–0.01	4%
Father is working	0.00	0.00	0.00	3%
Father's mental health scores	0.00	0.00	0.00	1%
Mother's mental health scores	0.00	0.00	0.00	0%
Residual	0.00	0.00	0.00	0%
Father attended high school	0.00	0.01	0.00	–2%
Child is male	0.00	0.00	0.00	–3%
Mother is working	0.00	0.00	0.00	–3%
Use improved drinking water	0.00	0.01	0.00	–3%
Total	–0.07	–0.07	–0.14	100%

## Discussion

Inequality in Indonesian children’s cognitive function favored more advantaged households in both 2000 and 2007. However, children aged 7–14 years in 2007 had higher cognitive scores than the cohort of 7–14 year olds in 2000. Importantly, although pro-rich inequality remains, inequality in cognitive function decreased by 45% between 2000 and 2007. Inequalities in per capita expenditures, maternal high school attendance and use of improved sanitation were the largest contributors to inequality for each year, suggesting that the change in cognitive function is most sensitive to these three important determinants. Children living in households with higher per capita expenditures, having a mother with high school education and using improved sanitation were more concentrated among the higher economic groups. Between 2000 and 2007, inequality in the social distribution of children with mothers who attended high school and used improved sanitation decreased. In these data, these were the main contributors to reduction in inequalities in children’s cognitive function. Our findings are consistent with the recent Program for International Student Assessment’s (PISA) results, indicating Indonesia was among the small number of countries where the level of socioeconomic inequality decreased and the average student’s performance improved between 2000 and 2009 [45].

**Figure 2:**
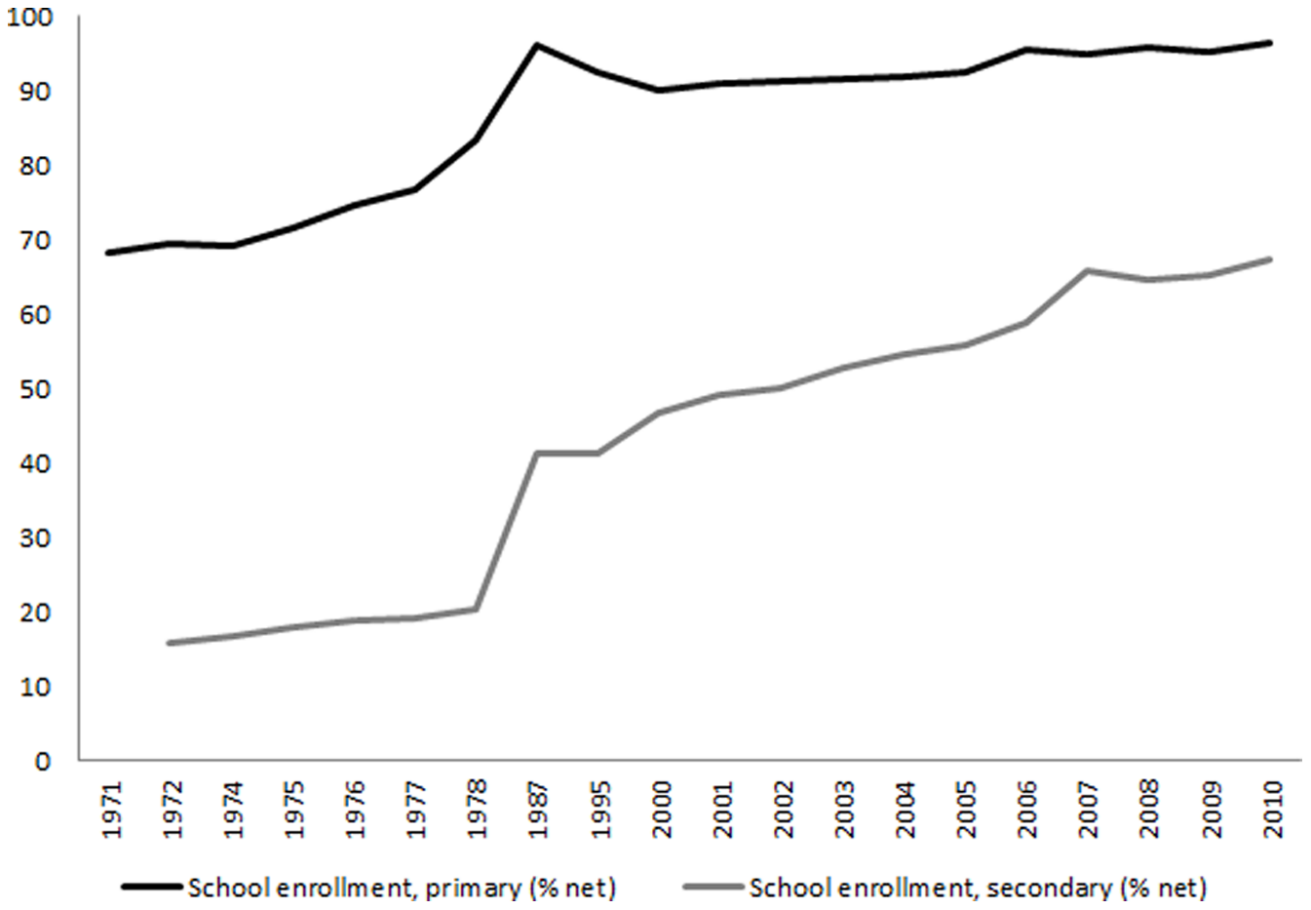
Trends in school enrolment, Indonesia, 1971–2010.

The decomposition analysis presented here is descriptive rather than causal, partly because we are limited by the factors included in the ILFS survey to examine as contributors. To triangulate our findings, we examined the national statistics on school participation, population with access to improved sanitation and GDP growth [46] as well as policy trends that may shed light on what we found to be the three largest contributors to reduction in cognitive function inequality. Statistics on trends in school participation show that between 1971 and 2010 both primary and secondary school enrolments have increased, with a substantial increase between 1971 and 1987 for primary school and between 1978 and 1987 for secondary school enrolment (Figure 2). One possible explanation is related to government policy to provide universal access to basic education through the school construction program in 1973–1979 and the elimination of primary school fees in 1978 [1]. Through the Presidential Instruction for Primary School program (*SD INPRES*), the government doubled the number of primary schools by building more than 61,000 new primary schools between 1973 and 1979. The number of schools constructed in each district was proportional to the number of primary school aged children not enrolled in schools in 1972 [47]. *SD INPRES* was not only the largest school construction program in the country’s history, but also the fastest increase in school provision in the world [1].A study by Duflo [47] suggested that this program had more impact on the cohort aged 7 or younger in 1974 but not the cohort born in 1962 or before. The same study also found this program had more impact in poor provinces and increased the probability of completing primary school by 12% among the affected children.

Using data from the IFLS 2000, Petterson [48] found that women and students from low socioeconomic groups received more benefits from the school construction program in Indonesia. Following that program, in 1984 the government passed the first law of compulsory basic education (*Wajib Belajar*), where every child aged 7–12 had to attend six years basic education.

**Figure 3:**
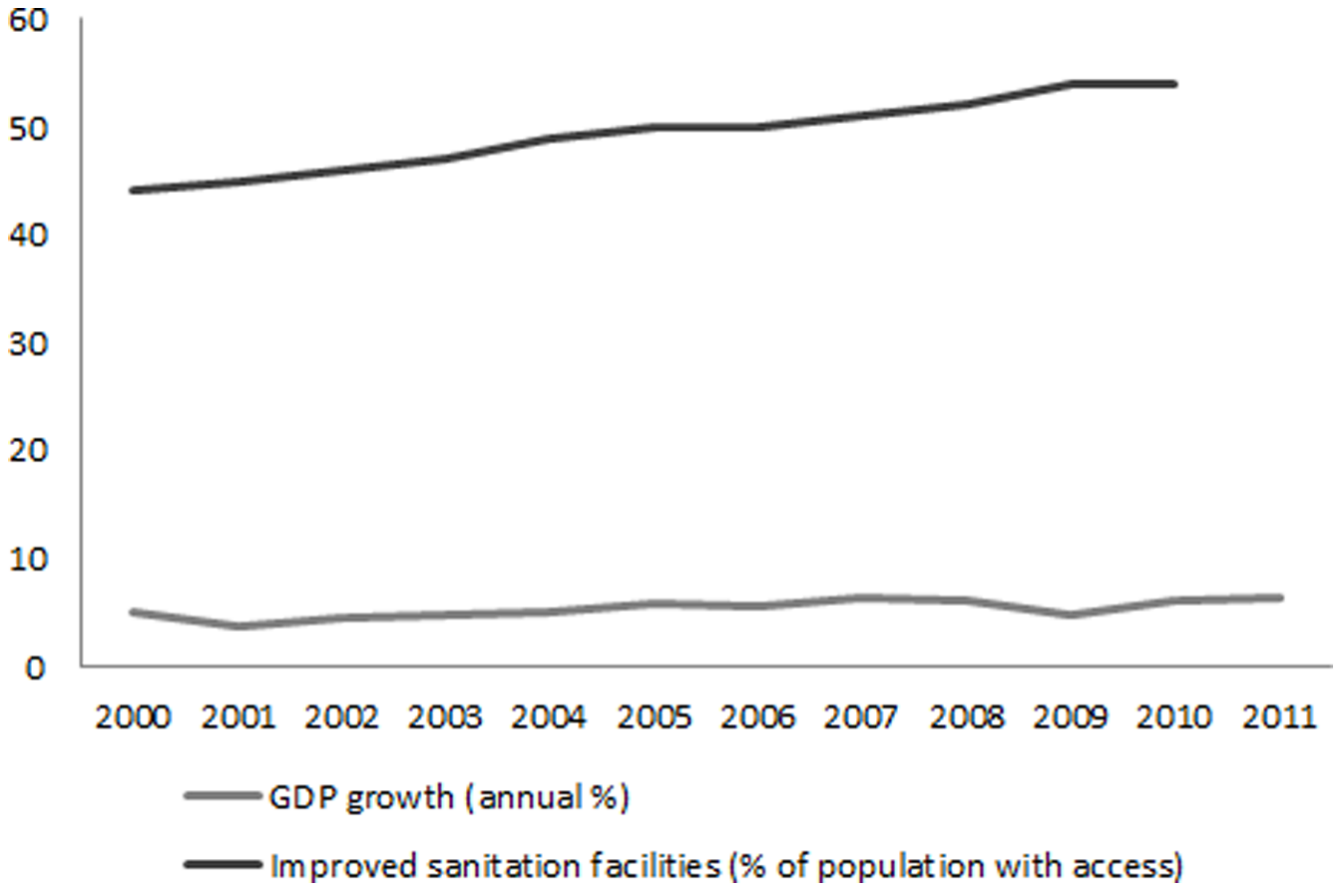
Trends in GDP growth and improved sanitation facilities, Indonesia, 2000-2011.

Our results show that reduction in the inequality in maternal high school attendance made the largest contribution to decreasing inequality in children’s cognitive function between 2000 and 2007. This is consistent with the evidence from developing countries that shows increasing school availability at the local level has greater benefit for educational achievement in females although the Indonesian program was not specifically targeting girls [54]. In our study, the mean age of mothers was 37 (SD 6.61 for 2000 and 6.58 for 2007). With the assumption that the cohort born after 1962 received more benefit from the school construction program, we estimated 85% of the mothers in 2007 were among the cohort who were more likely to benefit from the school construction program and the laws making six years basic education compulsory, compared to 55% of the mothers in 2000. In addition to change in inequality in maternal high school attendance, reduction in cognitive function inequality was also sensitive to change in household access to improved sanitation and change in per capita expenditures. Figure 3 shows steadily increasing trends in the proportion of the population with access to improved sanitation and improvements in the average GDP in 2000 and 2011.

A great deal of evidence shows that increasing pro-equity public service coverage including health and education programs could help reduce health related inequalities at the population level [49]–[51]. A systematic review by Gakidou, et al,[52] found increases in women’s educational attainment reduced gender gaps in education and contributed about 51% to decreasing under five mortality in 175 countries between 1970–2009. Evidence from Japan shows universal access to primary education in the early 1900s increased primary school attendance and reduced gender inequality in education, which in the longer term also had benefits on population health through reduction of infant mortality and increased life expectancy [53]. Qualitatively similar processes resulting in reductions in infant mortality on increasing life expectancy has also been demonstrated in South Korea [54].

The limitations of this study include that it is a descriptive analysis of cross sectional data in 2000 and 2007. Estimates of the magnitude and contribution to inequality calculated in these data are based on the socioeconomic measure used - in this case, per capita household expenditure. Other indicators of socioeconomic inequality may yield different estimates, especially for the contributions [41], [55]._ENREF_49Results from the decomposition analysis are sensitive to which determinants are selected for inclusion in the model. Remaining cognizant that our findings cannot be considered causal, we argue that pro-equity government policy and investment in education, particularly for women, improved sanitation and to a lesser extent economic growth are plausible important contributors to overall improvements and decreased inequalities in children’s cognitive function in Indonesia.
